# Digital Methods for the Spiritual and Mental Health of Generation Z: Scoping Review

**DOI:** 10.2196/48929

**Published:** 2024-02-06

**Authors:** Susanna Y Park, Bridgette Do, Jacqlyn Yourell, Janice Hermer, Jennifer Huberty

**Affiliations:** 1 Skylight Radiant Foundation Salt Lake City, UT United States; 2 Fit Minded Inc Phoenix, AZ United States; 3 Arizona State University Tempe, AZ United States

**Keywords:** Generation Z, Gen Z, spiritual health, digital mental health, spirituality

## Abstract

**Background:**

Generation Z (Gen Z) includes individuals born between 1995 and 2012. These individuals experience high rates of anxiety and depression. Most Gen Z individuals identify with being spiritual, and aspects from religion and spirituality can be integrated into mental health treatment and care as both are related to lower levels of depression. However, research on the spiritual and mental health of Gen Z is sparse. To date, there are no systematic or scoping reviews on digital methods to address the spiritual and mental health of Gen Z.

**Objective:**

This scoping review aimed to describe the current state of digital methods to address spiritual and mental health among Gen Z, identify the knowledge gaps, and make suggestions for how to leverage digital spiritual and mental health interventions for Gen Z.

**Methods:**

A comprehensive literature search was conducted in PubMed, Scopus, PsycInfo, CINAHL, Education Full Text, Google Scholar, SocIndex, and Sociological Abstracts. The inclusion criteria were as follows: (1) study population born between 1995 and 2012 (ie, Gen Z); (2) reporting on spiritual health or well-being, spirituality or religion, and mental health or well-being; (3) reporting on using digital methods; (4) publication in 1996 or beyond; (5) human subject research; (6) full text availability in English; (7) primary research study design; and (8) peer-reviewed article. Two authors screened articles and subsequently extracted data from the included articles to describe the available evidence.

**Results:**

A total of 413 articles were screened at the title and abstract levels, of which 27 were further assessed with full text for eligibility. Five studies met the inclusion criteria, and data were extracted to summarize study characteristics and findings. The studies were performed across 4 different countries. There were 2 mixed-methods studies (South Africa and Canada), 2 cross-sectional studies (China and United States), and 1 randomized controlled trial (United States). Of these studies, only 2 discussed digital interventions (a text messaging–based intervention to improve spiritual and mental health, and a feasibility study for a mental health app). Other studies had a digital component with minor or unclear spiritual and mental health measures. Overall, there was a lack of consistency in how spiritual and mental health were measured.

**Conclusions:**

Few studies have focused on assessing the spiritual and mental health of Gen Z in the digital context, and no research to date has examined a digital spiritual and mental health application among Gen Z. Research is needed to inform the development and evaluation of approaches to address the spiritual and mental health of Gen Z via digital means (eg, mobile apps).

## Introduction

The digital age is marked by widespread internet use and the ability to quickly communicate and find information online. Individuals born from 1995 to 2012 are considered “digital natives,” as they are the first generation to live in an age where technology and the internet are accessible at all times [1]. The Pew Research Center considers these individuals as part of Generation Z (“Gen Z”) and identifies the beginning of this generation to be in 1997 [1]. For the purpose of our research, we used the definition of Gen Z by Katz et al [2], who defined Gen Z as individuals born from 1995 to 2012, to account for those born when the World Wide Web made its public debut in 1995.

As a generation that grew up with technology, digital devices are familiar and seamlessly woven into the daily routines of Gen Z. It has been reported that 95% of Gen Z individuals have access to a smartphone [3]. In a study of 1000 Gen Z individuals aged 13 to 25 years, more than half spent 4 or more hours online compared with just 28% of all US adults who spent 4 or more hours online [4]. In a world where it is nearly impossible to socialize, work, and get an education without technology, Gen Z individuals are “always on,” and this is associated with higher rates of depression, attention deficit disorder or attention deficit hyperactivity disorder, and technology addiction [5]. Compared with other generations, Gen Z individuals spend more time alone or on digital communication platforms than engaging in in-person interactions. Between 2000 and 2015, Gen Z high school seniors spent an hour less on in-person social interactions compared with early millennials [6]. Adolescents who spend more time on social media than in in-person interactions are the loneliest compared with those who spend less time on social media. Moreover, between 2007 and 2018, there were great increases in the relative percentage rates for self-injury (47%), seriously considering suicide (76%), and suicide attempts (58%) among Gen Z [7].

The American Psychological Association (APA) states that Gen Z individuals are more likely to report mental health concerns (eg, depression and anxiety) than previous generations [8]. In a 2022 survey of 1055 Gen Z adults, 1 out of 4 reported having more bad days than good within a 1-month time frame. More than 2 out of 5 (42%) had a diagnosed mental health condition, with more than a quarter of those being diagnosed during the COVID-19 pandemic (March 2020) or later. Anxiety and depression are the 2 largest mental health issues among Gen Z, with 9 out of 10 individuals diagnosed with a mental health condition having anxiety and 8 out of 10 having depression [3]. Notably, Gen Z individuals are the most comfortable discussing their mental health [3,8]. One third of Gen Z individuals report posting about their mental health on social media. They also attend therapy and are willing to pay out of pocket for mental health care and services [3]. Despite the comfort of Gen Z in talking about their mental health, there is a crucial need to address the high rates of anxiety, depression, and other health issues that they experience [8].

Spirituality may be an untapped resource to address the mental health crisis experienced by Gen Z today. While spirituality can serve as a component within organized religion, the 2 aspects are distinct. Religion is an organized belief or specific set of practices focusing on a higher power (ie, Christian, Muslim, Buddhist, etc) [9]. Spirituality is a broader concept in which individuals seek connection to self, others, nature, and a sacred or higher being [10]. Individuals may identify with being either religious or spiritual, or both. Gen Z individuals do not necessarily identify with a particular religion or belief but instead practice spirituality. Only half of Gen Z individuals report turning to their faith for support in times of uncertainty [11], and they are more likely to engage in spiritual practices than religious practices [12]. In a study of 10,000 Gen Z individuals aged 13 to 25 years, 68% considered themselves religious and 77% considered themselves spiritual [13]. Gen Z individuals define spirituality as autonomous and faith unbundled, and it is inclusive of all faiths and practices [11,13].

Spirituality is related to several positive health and psychosocial outcomes, namely greater mental health [14]. A recently updated review of the literature on the relationship between spirituality and mental health found that greater spirituality was associated with lower depressive symptoms, lower suicidality, and lower substance abuse [14]. Gen Z individuals face some of the highest rates of mental health conditions (eg, depression) [15,16]; thus, spirituality should be considered in addressing youth mental health today. In the aforementioned report by Singer [13], the majority of Gen Z individuals attributed their spiritual connection to their positive mental health state. Another aspect of mental health that is influenced by spirituality is quality of life among chronically and terminally ill patients. Palliative care patients who struggle with spirituality report poorer quality of life compared with those who feel stable with their spirituality [17]. Additionally, teens and young adults with cancer mention searching for meaning, hope, and life perspectives, even though they may not consider themselves as spiritual [18]. Interventions that promote spiritual well-being (one’s sense of purpose, meaning in life, and connection to something greater [19]) may be a powerful resource for improving mental health in Gen Z.

Research on digital mental health interventions and spirituality exist separately. Little is known about digital methods (eg, mobile apps, text messaging, etc) that incorporate both spiritual and mental health among Gen Z. Scoping or systematic reviews on this topic are nonexistent, and research on this topic is very limited. Given that technology is woven into the daily lives of Gen Z, digital mobile apps that promote spirituality may offer a novel approach to supporting the mental health of Gen Z adolescents and young adults. Therefore, the purpose of this scoping review was to describe the current state of digital methods to address spiritual and mental health among Gen Z, identify the knowledge gaps, and make suggestions for how to leverage digital spiritual and mental health interventions for Gen Z.

## Methods

### Eligibility Criteria

The inclusion criteria for targeted articles were as follows: (1) study population born between 1995 and 2012 (ie, Gen Z); (2) reporting on spiritual health or well-being, spirituality or religion, and mental health or well-being; (3) reporting on using digital methods; (4) publication in 1996 or beyond; (5) human subject research; (6) full text availability in English; (7) primary research study design; and (8) peer-reviewed article.

### Information Sources

Guided by the Preferred Reporting Items for Systematic Reviews and Meta-Analyses (PRISMA) extension for scoping reviews (PRISMA-ScR) [20], searches were conducted in PubMed, Scopus, PsycInfo (EBSCO), CINAHL Plus with Full Text (ProQuest), Education Full Text (H.W. Wilson), Google Scholar, SocIndex with Full Text, and Sociological Abstracts (ProQuest) on May 2 and 3, 2023, by a health sciences librarian (J Hermer) at Arizona State University. The same information sources were searched on September 21-26, 2023, and again on November 7-8, 2023, and were also included in the results. ERIC and Atla Religion Database were also searched, but they returned no results.

### Search Strategy

The searches were optimized for each individual database but included a combination of keywords and subject headings for the following 4 categories: Generation Z or Gen Z; spirituality or religion; mental well-being; and mobile health, mHealth, and eHealth (Multimedia Appendix 1). Owing to the very limited nature of the results, only language filters were applied to ensure that all relevant literature was available to be screened. All records were imported into Zotero to check if any articles were retracted and then into Covidence systematic review software for deduplication and screening [21]. Apart from the initial search, 2 additional searches were conducted to ensure that all relevant literature was found and to add any recent literature from the following 6 months. A search of all databases was performed on September 21-26, 2023, using the additional keywords of “faith,” “transcenden*,” and “life purpose” or “existential needs,” and a final search was performed on November 7, 2023, using the additional keywords of “spiritu*” and “relig*.” The scoping review protocol was registered online in Open Science Framework (OSF) on May 16, 2023.

### Selection Process

The selection process was completed entirely on Covidence. Prior to reviewing titles and abstracts, duplicate titles were eliminated by Covidence. Two authors (SYP and BD) screened all titles and abstracts independently and were blinded to each other’s decisions. Any disagreements were discussed between the authors SYP and BD, and agreed upon before full-text review. Agreement scores for article selection between the 2 authors were not logged in Covidence for the initial search; however, the agreement scores were 85% (81/95) and 90% (80/87) for reviewing titles and abstracts between the 2 authors for the September and November searches, respectively. For full-text article review, the author SYP independently reviewed half of the articles, and the author BD independently reviewed the remaining articles. The authors SYP and BD deliberated with each other, and with J Huberty and JY if there were any questions regarding inclusion based on the article eligibility criteria. Articles were excluded if the study population included Gen Z but did not explicitly distinguish Gen Z in the population sample and the results were not disaggregated by age. The final articles included in the review were agreed upon by all 4 authors.

### Data Collection Process and Synthesis Methods

Prior to the search, all authors agreed on the following characteristics for data extraction and synthesis: title; authors; study country; study objectives; study design; data collection timeframe; recruitment methods; sample size; participant characteristics; description of digital methods; constructs related to religion, spirituality, or spiritual well-being; assessment or measure for religion, spirituality, or spiritual well-being; constructs related to mental health; assessment or measure for mental health; main findings; and study limitations. These characteristics were selected to ensure a detailed understanding of available literature as it relates to the goal of the scoping review. For articles included in the scoping review, the author SYP independently extracted data based on the a priori characteristics for half of the articles and the author BD independently extracted data from the remaining articles. Given that each article had data extracted by a single author, there were no agreement scores. After evaluating the included articles, study characteristics and main findings were summarized in a descriptive manner.

Some studies that initially appeared eligible for this review were ultimately excluded because they did not meet specific criteria. For instance, a cross-sectional study on the perceptions of 475 Gen Z individuals and young millennials and their use of a spiritual self-care app [22] was originally included when reviewing titles and abstracts. However, upon full-text review, we found that the study results did not distinguish between Gen Z individuals and young millennials, thus failing to meet our review’s criteria (ie, Gen Z only).

## Results

### Study Characteristics

**Figure 1:**
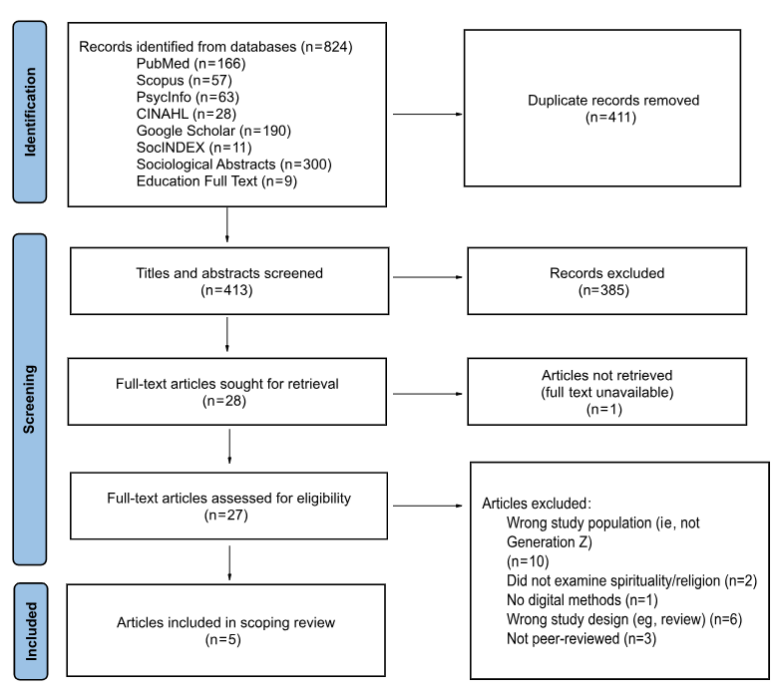
PRISMA (Preferred Reporting Items for Systematic Reviews and Meta-Analyses) flow diagram of article selection.

Details on article selection are illustrated in a PRISMA diagram (Figure 1). Our search identified 824 articles from 8 databases based on the search terms. After removing 411 duplicates in Covidence, 413 articles were screened by title and abstract. After the initial screening based on the inclusion criteria, the full texts of the remaining 27 articles were screened. Review articles were not included in the final review, but references were screened to see if any additional literature was admissible. Ultimately, the scoping review included 5 articles. Of these 5 articles, 1 [23] was included in the review from the updated search conducted in September 2023 and 1 [24] was included from the updated search in November 2023. Characteristics and results of the studies are summarized in Table 1.

**Table 1:** Characteristics and main findings of the included studies.

Reference and study country	Study design	Sample characteristics	Digital aspect^a^	Religion/spirituality measures^b^	Mental health measures^b^	Main findings
Mindu et al [25], 2023 (South Africa)	Mixed-methods study	93 youth and young adults aged 16-24 years; 44% female	Assessed participants’ knowledge and preferences for a digital mental health app	Where do youth seek treatment or assistance when they have mental health problems (Response options: Visit a spiritual healer; Go to a church for prayers; Traditional medicine; Clinic/hospital; Visit a health care worker)	Mental health awareness (eg, taught about mental health and no prior education on mental health) Mental health conditions affecting youth in the community (eg, substance abuse or misuse, posttraumatic stress disorder, depression, and anger)	No participants had experience using a mental health app, but 99% indicated mental apps are important and can benefit youth. Religious and cultural beliefs were a barrier to using digital platforms (eg, social media).
Gao et al [26], 2021 (China)	Cross-sectional study	1017 first-year college students (mean age 19 years); 77.8% female	Participants were recruited through an eHealth application to complete a survey	Religion (Response options: No religion; Buddhism; Christian; Others)	Generalized Anxiety Disorder-7 (GAD-7) and depression; Patient Health Questionnaire-9 (PHQ-9) [27,28]	95.3% indicated having no religion. Belief in Christianity and in Buddhism were associated with greater anxiety.
Craig Rushing et al [29], 2021 (United States)	Randomized controlled trial	833 American Indian or Alaska Native teenagers and young adults aged 15-24 years; 66.3% female	The 2 intervention arms included 3 SMS text messages per week for 8 weeks with information, role model videos, images, and engagement opportunities (eg, reply for more information, resource links, etc)	Rate your spiritual health (Response options: Excellent; Very good; Good; Fair) [30]	Rate your mental health (Response options: Excellent; Very good; Good; Fair) [30]	No significant differences between the 2 intervention arms within subjects. Mean scores of perceived health (physical, mental, and spiritual) significantly increased over time for both intervention arms. Those who reported better health also reported greater cultural resilience, identity, and cultural pride.
Reed et al [23], 2022 (United States)	Cross-sectional study	349 American Indian or Alaska Native youth aged 15-24 years; 71.1% female	Assessed participants’ use of media technologies (ie, media types, frequency, and duration) and how they use media technologies (ie, online behaviors and activities)	Select the top 3 health topics from a list of 15, including spiritual health	Self-reported mental health (How good is your mental health? Response options: 4-point Likert-type scale; 4=Excellent and 1=Poor) Select the top 3 health topics from a list of 15, including mental health	53.5% of participants relied heavily on the internet to access health information. Nonsexual and gender minority participants reported better mental health than sexual and gender minority participants. The top 3 most important health topics were Native identity, mental health, and social justice and equality. Spiritual health was selected as the most important health topic 14% of the time.
Au-Yeung et al [24], 2023 (Canada)	Mixed-methods study	5 Indigenous youth aged 11-16 years; 60.0% female	Assessed participants’ opinions on the JoyPop app, and the accessibility and feasibility of the app	Qualitative question: “What does a ‘Good Mind’ (Haudenosaunee concept) mean to you?” “The Good Mind is a physical, psychological, and spiritual journey that includes a reflective awareness of thoughts and intentions, and a way of being that is expressed through self-compassion and compassion for other beings.”	Perceived mental wellness (5-point Likert scale) Qualitative question: “Would you please describe what mental wellness means to you?”	Participants had mixed ratings for their mental health from “fair” to “very good.” Participants reported nature to be important for a “Good Mind.” Participants reported enjoying the app’s features (eg, games and breathing techniques) and made suggestions to make it relevant to their culture.

^a^Refers to the digital component of the study, which may include the mode through which an intervention was given (eg, mobile text messages) or the constructs measured (eg, social media use and preferences for a mobile app).

^b^Measures refer to assessments, tools, or specific survey items that were used in the study to assess the construct of interest (eg, religion or spirituality, and mental health).

### Study Design and Study and Sample Characteristics

All 5 studies examined Gen Z (adolescents and young adults; aged 11-24 years; born between 1995 and 2012). Of the 5 articles, 2 were published in 2023, 1 was published in 2022, and 2 were published in 2021. There were 2 mixed-methods studies (1 in South Africa [25] and 1 in Canada [24]), 2 cross-sectional studies (1 in China [26] and 1 in the United States [23]), and 1 randomized controlled trial in the United States [29]. Sample sizes varied across the 5 studies, ranging from 5 to 1017.

#### Study by Mindu et al, 2023

Mindu et al [25] conducted a mixed-methods study in South Africa to inform the implementation of a mobile phone–based mental health intervention [25]. The study did not use any specified measures for mental health but asked questions via quantitative and qualitative methods to understand participants’ understandings or perceptions of mental health. Questions addressed (1) mental health awareness; (2) digital interventions for mental health; (3) access to digital devices and the internet; (4) preferences for digital mental health interventions; and (5) barriers to the use of digital mental health innovations. Quantitative results showed that almost half (49%) of the participants had heard about mental health apps, none had experience using one, and 99% indicated that mental health apps are important and can benefit youth. These results underscore the severe lack of mobile mental health apps designed for individuals in South Africa. Qualitative results revealed that religious and cultural beliefs were a barrier to using digital platforms (eg, social media), and the authors highlighted the crucial need to develop culturally appropriate and relevant digital apps that represent those who they serve. Overall, participants expressed high interest in using a digital mental health app (eg, social media) to learn about mental health and seek resources. The study authors acknowledged that while no digital methods or interventions were implemented in the study, the results provided a unique Gen Z perspective on the usability of digital methods.

#### Study by Gao et al, 2021

Gao et al [26] conducted a cross-sectional study in China among first-year Chinese college students. Using a health management app (Residents e-Health), a questionnaire was distributed to examine depression and anxiety, and their associations with other health-related constructs, such as stress and nutrition. The authors examined participants’ religious beliefs (ie, Christian, Buddhism, other, and no religion) as a correlate of anxiety and depression. However, most of the sample (95%) did not have a religion. Belief in Christianity and belief in Buddhism were associated with greater anxiety in the sample. The study authors noted that the large number of participants ascribing to no religion limited the understanding of the relationship between religion and mental health. The overall conclusion of the study indicated that early lifestyle interventions assessing religion, as well as other demographic and behavioral factors, are important for understanding the factors contributing to mental health in Gen Z.

#### Study by Craig Rushing et al, 2021

Craig Rushing et al [29] conducted a randomized controlled trial in the United States to examine the efficacy of an mHealth intervention (BRAVE) for physical, mental, and spiritual health; resilience; self-esteem; and coping and help-seeking skills among American Indian or Alaska Native teenagers and young adults. Participants were randomized to participate in 1 of 2 groups: (1) An 8-week intervention arm involving 3 SMS text messages per week highlighting common coping strategies, preferred wellness strategies, help-seeking skills, and related protective factors such as cultural resilience, identity, and cultural pride; or (2) An 8-week control arm involving 3 SMS text messages per week designed to elevate and reaffirm Native voices in STEM (science, technology, engineering, and mathematics) and medicine. Participants were in both arms, and both had messaging that included a combination of information, role model videos, images, and opportunities of engagement (eg, reply for more information and links to access resources). The findings indicated that there were no significant differences between the 2 study arms within subjects, such that participants in the intervention arm did not report better outcomes than those in the control arm. However, mean scores of perceived health (ie, physical, mental, and spiritual) significantly increased over time in both arms. In addition, participants who reported better health also reported greater cultural resilience, identity, and cultural pride. The percentage of participants who used the resources and information in the intervention arm text messages also increased over 5 months. The study authors acknowledged some limitations, including high or favorable survey measure outcomes at baseline, only a 1-week break between receiving interventions in the arms, and control messaging likely being novel and helpful. Another limitation to note is the assessment of perceived health, which combined 3 separate survey measures assessing perceived physical, mental, and spiritual health. The combination of 3 different aspects of health makes it challenging to derive valid inferences regarding mental or spiritual health on their own. Overall, the intervention demonstrated improved health outcomes and underscores the acceptability of text messaging to promote and support well-being.

#### Study by Reed et al, 2022

Reed et al [23] conducted a cross-sectional study in the United States, in which 349 American Indian or Alaska Native youth (aged 15-24 years) were asked about the extent to which they use media technologies, how they use technologies, and their health priorities. Several trends were revealed. The majority of participants (64.7%) reported sending 1 to 50 text messages a day. Instagram was the most popular daily technology used, and 65.3% of participants reported using social media 3-7 hours per day. Participants also self-rated their mental health. The findings indicated that nonsexual and gender minority youth (56.7%) reported better mental health than sexual and gender minority youth (36.4%). To better understand important health topics, participants were asked to select their top 3 health topics from a list. The most popular topic selected was Native identity or cultural pride (73%), followed by mental health (57%) and social justice and equality (31%). Spiritual health was selected by 14% of youth in their top 3 health topics. While spiritual health was not among the top health topics selected, it is important to note that the range of options offered spanned across several categories (eg, social justice or inequality, alcohol and drug use or abuse, and the environment). Overall, the study authors concluded that building resources that foster cultural pride and positive identity must be included in any programs or technologies for addressing mental health among American Indian or Alaska Native youth.

#### Study by Au-Yeung et al, 2023

Au-Yeung et al [24] conducted a Haudenosaunee (Canada’s largest First Nations reserve, Six Nations of the Grand River) community-based study in which 5 Haudenosaunee youth (aged 11-16 years) tested the JoyPop mobile app (available in English and French) that is designed to promote resilience among youth. The app offers breathing exercises, mood tracking, journaling, personalizable social support, a 24-hour helpline, and games. The results indicated that participants had mixed ratings on their self-reported mental wellness, ranging from “fair” to “very good,” and that they used the app 1 to 3 times a day. Of the 5 participants, 4 were interviewed about their experiences using the app, perspectives of mental wellness, and characteristics of a “Good Mind” (an Indigenous concept on the physical, psychological, and spiritual journey that maintains balance and harmony in a person). Interview participants reported that the app was easy to use and esthetically pleasing. They also enjoyed all of the app’s features, with the exception of the Circle of Trust feature (ie, personalizable social support). Interview participants identified positivity and happiness, understanding emotions, acts of kindness, personal hobbies, and positive body language as important to their mental wellness. Participants also discussed important characteristics of a Good Mind, such as positivity, kindness, and connecting with nature. Overall, the app was favorable to the participants, but they suggested incorporating specific features like words in their own language and Indigenous visuals (eg, feathers and clan animals). While the app lacked explicit content on a Good Mind, the authors recommended incorporating concepts of a Good Mind to enhance its relatability to Haudenosaunee youth, given its cultural significance to the Haudenosaunee people. Although the study had a small sample size, the authors concluded that mobile health interventions can be beneficial to Indigenous youth, as mental health apps continue to be of interest and Indigenous cultures value the promotion of health and resilience. Further, Indigenous tribes across North America have unique perspectives, and pan-Indigenous resilience apps like JoyPop will need to be tailored to specific cultural contexts.

## Discussion

### Overview

The purpose of this scoping review was to describe the current state of digital methods to address spiritual and mental health among Gen Z, identify the knowledge gaps, and make suggestions for how to leverage digital spiritual and mental health interventions for Gen Z. A comprehensive literature search across 8 databases identified only 5 relevant studies, emphasizing the significant lack of published research on digital methods to address the spiritual and mental health of Gen Z. Among the 5 studies, only 2 discussed digital interventions, and of these studies, 1 examined a text messaging–based intervention to improve spiritual and mental health and reported improvements in spiritual and mental health over time [27], and 1 examined the feasibility of a mental health app [24]. The sparse available literature limits conclusions on the current state of digital methods to address spiritual and mental health, and warrants future research to address these gaps.

### Current State of Digital Methods for Spiritual and Mental Health

Gen Z individuals are facing a mental health crisis as they experience high rates of depression and anxiety [8,16]. For example, one of the included studies reported that the prevalences of anxiety and depression among college freshmen were 40.3% and 45.3%, respectively [26]. Gen Z individuals also report feeling lonely, having low self-confidence, and being distressed about the future [3,5,12]. All 5 studies included in this review discussed the overall well-being of Gen Z and, to some extent, aspects of spirituality. The findings suggest that spirituality may play a role in the mental health of Gen Z and should be considered in the development and implementation of future digital applications that address mental health. The study by Mindu et al [25] particularly underscores the need for customizability in mental health applications considering that participants expressed that using digital platforms (ie, social media) conflicted with their religious and cultural beliefs. Thus, the introduction of applications that allow users to engage in practices and view content that aligns with their values and beliefs is a potential avenue for combating this barrier and could in turn strengthen one’s spirituality. However, research is needed to determine the acceptability of digital mental health applications that involve spiritual content.

Nearly every facet of the lives of Gen Z involves technology, for example, using computers for school or work, using mobile apps to order food, and using social media and texting to communicate with friends. Despite the widespread use of technology among Gen Z and the increasing number of research studies employing digital methods to test and deliver mental health programs and interventions [31], only 1 of the studies included in this review examined the effects of a digital intervention on spiritual and mental health [27] and 1 assessed the feasibility of a mental health mobile app [24]. The lack of digital methods or interventions to address the spiritual and mental health of Gen Z warrants the development of accessible ways for Gen Z to practice spiritual and mental self-care. Gen Z individuals spend more time (4 or more hours daily) on social media compared with other generations [3], and poor mental health is often attributed to social media use [32]. However, social media is not necessarily harmful and is instead dependent upon what Gen Z individuals do and see online, their pre-existing strengths or vulnerabilities, and the environment in which they are raised (eg, parental monitoring) [32,33]. Digital methods for spiritual wellness that are specifically targeted for Gen Z and built to empower Gen Z to practice self-care and build healthy coping mechanisms may benefit the mental health of Gen Z. Results from this review illustrate the acceptability of a mobile intervention promoting spiritual and mental health [24,29], and the interest Gen Z individuals have toward using digital methods to address their spiritual and mental health [25]. However, given that little is known about this topic, more research is needed to truly grasp the feasibility and efficacy of digital approaches to address spiritual and mental health with regard to the well-being of Gen Z.

### Gaps in the Literature

Published literature or research about the spiritual and mental health of Gen Z is limited, reporting mostly mental health statistics rather than examining determinants of mental health or interventions for improving mental health (eg, spirituality) [1,33]. Research to date on spirituality in Gen Z has only been performed by faith-based organizations or by nonacademic research institutions and has mainly focused on comparing the views of Gen Z on religion and spirituality to the views of other generations [11,12,34]. For example, the majority of Gen Z individuals (77%) identify as spiritual, preferring to ascribe to a set of values from various beliefs [13]. While we know that the majority of Gen Z individuals identify as spiritual, empirical research that specifically examines their spiritual practices and preferences, overall spiritual well-being, and associations with mental and physical health outcomes is warranted. Among the 5 studies included in this review, 3 examined spiritual health. The randomized controlled trial [29] asked participants to rate their spiritual health using a combined measure for overall health that included physical, mental, and spiritual health, limiting the ability to assess spiritual well-being explicitly among these participants. The cross-sectional survey [23] asked participants to select the top 3 health topics important to them (eg, mental health, spiritual health, and Native identity), but the study did not examine any associations between spiritual health and mental health. Spiritual health was not defined for participants in either study. The mixed-methods study in Canada [24] interviewed participants who used an app designed to promote resilience in youth with regard to a Good Mind, an Indigenous concept on the physical, psychological, and spiritual journey that maintains balance and harmony in a person. However, this concept is specific to the Haudenosaunee people; therefore, the findings may not be applicable to the perspectives on spirituality of other young communities. In addition, only 1 article administered a valid measure to assess mental health, spirituality, or spiritual well-being (ie, Generalized Anxiety Disorder-7 [GAD-7]) [26]. The validity and reliability of instruments used to measure mental health and spirituality or spiritual well-being in the study samples were not reported in any of the included articles. Identified gaps offer research opportunities to comprehensively examine spiritual and mental health among Gen Z.

### Suggestions for Leveraging Digital Spiritual and Mental Health Interventions for Gen Z

Gen Z individuals are remarkably familiar with navigating digital spaces and integrating spirituality into their lives, and are the most comfortable talking about their mental health compared with other generations [3]. Gen Z individuals who have a spiritual connection have better perceptions about their mental health and believe their spiritual health contributes to their mental health, compared with those who do not have a spiritual connection [11]. Since Gen Z individuals primarily consume information through technology, digital interventions are a promising method to teach and facilitate various practices in spiritual and mental self-care. For example, the digital intervention content of Craig Rushing et al [29] included wellness strategies, such as self-care and goal setting, which resulted in improved perceived health (eg, physical, mental, and spiritual) for participants. In the study by Mindu et al [25], digital mobile interventions through social media were concluded to be potentially useful to increase mental health literacy and knowledge of resources. Additionally, the study by Gao et al [26] suggested that early interventions that target the lifestyle behaviors of Gen Z (ie, smoking) can improve their depression and anxiety. A spiritual self-care app tailored for Gen Z, for example, may allow Gen Z to engage with full autonomy and convenience. Rather than requiring Gen Z to seek out places to practice religious or spiritual beliefs, a spiritual self-care app can be available wherever they are and whenever they want. Digital spiritual self-care interventions should also consider incorporating topics that are deemed important to Gen Z, such as cultural relevance, inclusivity, social justice, and nature [23,24].

Mobile apps grant users the ability to customize their experience, which vastly differs from traditional means of practicing religion that ascribe to a predetermined set of beliefs, values, and practices that are often fixed [13]. Access to technology enables Gen Z individuals to autonomously decide which aspects of different spiritual beliefs and practices they resonate with. Most digital apps include features that allow users to customize their experience and the content they engage with. Thus, a mental health app that includes various components of spiritual practices that users can choose from may provide a new way for young people to tend to their spiritual and mental health. Based on this review, research examining the feasibility, acceptability, and efficacy of digital health tools specifically targeting spiritual and mental health among Gen Z is absent, indicating the need for research in this area. Researchers, companies, and nonprofit organizations can leverage existing digital spaces that Gen Z individuals frequently use (eg, Instagram and TikTok) to garner feedback on what they might desire in a digital spiritual and mental health intervention. For example, 2 of the studies [24,29] incorporated feedback from Indigenous youth on existing digital methods (eg, SMS text messaging and JoyPop) to inform the development of their digital content. Spiritual well-being apps, for example, can be used to target Gen Z and deliver evidence-based content that integrates spirituality and mental health. These studies also emphasize the importance of culturally relevant interventions that can speak to diverse cultural backgrounds and beliefs among Gen Z.

### Strengths and Limitations of This Review

There are multiple strengths of this review. First, this scoping review indicates that there is a limited knowledge base surrounding digital methods for addressing the spiritual and mental health of Gen Z and summarizes the current state of the literature on this topic. This is the first scoping review to address this topic and highlights a crucial gap in supporting young people’s mental health. Additionally, a librarian was consulted and involved in the search process to bolster the rigor and accuracy of the review. Covidence was used to minimize human error in screening eligible articles. Along with these strengths, there are some limitations in this review. First, a scoping review limits the objective understanding of a topic such that quantitative results cannot be compiled to determine effect sizes across studies. Second, database searches are not uniform and require nuanced search methodologies, which can result in relevant studies being missed. Third, manuscripts during the full-text screening stage were split between the authors SYP and BD, and thus, manuscripts were assessed by a single rater. Finally, research on this topic is severely lacking, which limits the number of articles included in this review and impacts the ability to construct a cohesive narrative or draw definitive conclusions about the state of the field. This underscores the necessity of this review to highlight the gaps and urge further investigation.

### Scientific Contribution

The goal of this scoping review was to assess the extent to which research on digital methods to address the mental and spiritual health of Gen Z has been conducted. The scoping review revealed a lack of available research on spirituality and mental health. Specifically, there is a dearth of studies on the use of digital methods to deliver spiritual well-being for mental health in Gen Z. Despite an increase in mental health concerns among Gen Z and the growing body of evidence on the beneficial effects of spiritual self-care on mental health, few published articles touch on this topic. The findings from this review highlight the opportunity for addressing the mental and spiritual health of Gen Z through digital methods (eg, mobile apps). The use of digital methods to address mental health is a growing area of research; however, spirituality and spiritual self-care have received little attention. There is potential for researchers to examine spiritual self-care, which is delivered through digital methods, and its impact on populations experiencing significant mental health problems. Overall, the scoping review underscores the need for future research to examine the acceptability and feasibility of digital approaches to address spiritual and mental health among Gen Z.

### Conclusion

This scoping review underscores the dearth of research surrounding digital methods to address spiritual and mental health among Gen Z. Considering that digital methods to address aspects of mental health are increasingly popular and effective [35,36], research is needed to examine digital platforms that address spiritual and mental health. This is especially pertinent for Gen Z individuals as they have some of the greatest rates of mental health issues and are the most digitally savvy generation to date, and most indicate that they are spiritual. Leveraging spirituality as a way to address mental health among Gen Z via digital means offers a novel and relevant approach for addressing the mental health crisis impacting young people today.

## Appendix

Multimedia Appendix 1 Databases and search terms used for review.

Multimedia Appendix 2 PRISMA (Preferred Reporting Items for Systematic Reviews and Meta-Analyses) checklist.

## Abbreviations

Gen Z: Generation Z
PRISMA: Preferred Reporting Items for Systematic Reviews and Meta-Analyses

## Data Availability

The full review process, including the search terms and databases used, is available as a supplementary file.
