# Supplementary material for: Digital Methods for the Spiritual and Mental Health of Generation Z: Scoping Review
Source: Interact J Med Res. 2024 Feb 6;13:e48929. doi: 10.2196/48929 (PMC10879969; doi:10.2196/48929)
Supplement: Multimedia Appendix 1 [file ijmr_v13i1e48929_app1.docx]

**Multimedia Appendix 1.** Databases and search terms used for review.

**PubMed = 47 results**

(((Gen Z OR "generation Z" OR youth OR teens OR teenager* OR "young adult" OR "emerging adult" OR "college students" OR "university students" OR adolescent* OR adolescence OR post-millennials”) AND (mental health[MeSH Terms] OR (mental AND (wellness OR wellbeing OR well-being OR health)) OR depression OR anxiety OR stress)) AND (Spirituality[MeSH Terms] OR "spiritual well being" OR Spirituality OR spiritual health OR religiosity OR religion[MeSH Terms] OR relig* OR religion OR Christian* OR Islam OR Muslim OR Buddhism OR Buddhist OR Hindi OR Hinduism OR Jewish OR Judaism OR Sikh)) AND ((mhealth OR "digital health" OR "mobile app*" OR "mobile health" OR "digital intervention" OR ehealth OR telehealth OR "virtual medicine" OR telehealth OR "health app") OR (telehealth[MeSH Terms]))

Updated Sept.21, 2023 = 57 results

((("Gen Z" OR "generation Z" OR youth OR teens OR teenager* OR "young adult" OR "emerging adult" OR "college students" OR "university students" OR adolescent* OR adolescence OR "post-millennials") AND (mental health[MeSH Terms] OR (mental AND (wellness OR wellbeing OR well-being OR health)) OR depression OR anxiety OR stress)) AND (Spirituality[MeSH Terms] OR "spiritual well being" OR Spirituality OR spiritual health OR spiritu* OR religiosity OR religion[MeSH Terms] OR relig* OR religion OR Christian* OR Islam OR Muslim OR Buddhism OR Buddhist OR Hindi OR Hinduism OR Jewish OR Judaism OR Sikh OR "life purpose" OR transcenden* OR faith OR "Existential needs")) AND ((mhealth OR "digital health" OR "mobile app*" OR "mobile health" OR "digital intervention" OR ehealth OR telehealth OR "virtual medicine" OR "health app") OR (telehealth[MeSH Terms])) Filters: English

**Updated Nov.7, 2023 = 62 results**

((("gen z" OR "generation z" OR youth OR teens OR teenager* OR "young adult" OR "emerging adult" OR "College students" OR "university students" OR adolescent* OR adolescence OR "post-millennials") AND (((mental health[MeSH Terms]) OR (mental AND (wellness OR wellbeing OR "well being" OR health))) OR (depression OR anxiety OR stress))) AND (((spirituality[MeSH Terms]) OR (religion[MeSH Terms])) OR (spiritu* OR relig* OR "spiritual well being" OR "spiritual wellbeing" OR spirituality OR "spiritual health" OR religiosity OR religion OR Christian* OR Islam OR Muslim OR Buddhism OR Buddhist OR Hindi OR Hinduism OR Jewish OR Judaism OR Sikh OR "life purpose" OR transcenden* OR faith OR "existential needs"))) AND ((mhealth OR "digital health" OR "mobile app*" OR "mobile health" OR "digital intervention" OR ehealth OR telehealth OR "virtual medicine" OR "health app") OR (telehealth[MeSH Terms]))

Filters: English, articles self-limit to 2013

**Scopus = 17 results**

mhealth OR "digital health" OR "mobile app" OR "mobile health" OR "digital intervention" OR ehealth OR telehealth OR "virtual medicine" OR telehealth OR "health app"

AND

"gen z" OR "generation Z" OR youth OR teen* OR "young adult" OR "emerging adult" OR "college students" OR "university students" OR adolescent* OR adolescence

AND

"mental health" OR wellness OR wellbeing OR depression OR anxiety OR stress

AND

spirituality OR spiritual OR religiosity OR religion OR christian OR christianity OR islam OR muslim OR buddhism OR buddhist OR hindi OR hinduism OR jewish OR judaism OR sikh

Updated search, Sept.21, 2023 = 18 results

mhealth OR "digital health" OR "mobile app" OR "mobile health" OR "digital intervention" OR ehealth OR telehealth OR "virtual medicine" OR telehealth OR "health app"

AND

"gen z" OR "generation Z" OR youth OR teen* OR "young adult" OR "emerging adult" OR "college students" OR "university students" OR adolescent* OR adolescence

AND

"mental health" OR wellness OR wellbeing OR depression OR anxiety OR stress

AND

faith OR spirituality OR spiritual OR religiosity OR religion OR christian OR christianity OR islam OR muslim OR buddhism OR buddhist OR hindi OR hinduism OR jewish OR judaism OR sikh OR "life purpose" OR transcend* OR "Existential needs"

Filters: English only, 2003 onwards

**Updated search Nov.7, 2023 = 22 results**

mhealth OR "digital health" OR "mobile app" OR "mobile health" OR "digital intervention" OR ehealth OR telehealth OR "virtual medicine" OR telehealth OR "health app"

AND

"gen z" OR "generation Z" OR youth OR teen* OR "young adult" OR "emerging adult" OR "college students" OR "university students" OR adolescent* OR adolescence

AND

"mental health" OR wellness OR wellbeing OR depression OR anxiety OR stress

AND

spiritu* OR relig* OR faith OR spirituality OR spiritual OR religiosity OR religion OR christian OR christianity OR islam OR muslim OR buddhism OR buddhist OR hindi OR hinduism OR jewish OR judaism OR sikh OR "life purpose" OR transcend* OR "Existential needs"

Filters: English only, 2003 onwards

**PsycInfo (ProQuest) = 18 results**

(mainsubject(spiritual well being) OR mainsubject(spirituality) OR "spiritual health" OR (spiritual* OR religion OR religiosity) OR (christian OR christianity OR islam OR muslim OR buddhism OR buddhist OR hindi OR hinduism OR jewish OR judaism OR sikh)) AND (subject(mental health) OR tiab(mental wellness OR mental wellbeing OR depression OR anxiety OR stress)) AND (("generation Z" OR "gen Z" OR youth OR "young adult" OR adolescent OR teenager OR "emerging adult" OR teen* OR "college students" OR "university students") AND (mhealth OR "digital health" OR "mobile app*" OR "mobile health" OR "digital intervention" OR ehealth OR telehealth OR "virtual medicine" OR telehealth OR "health app"))

Updated seach, Sept.21, 2023 = 22 results

(mainsubject(spiritual well being) OR mainsubject(spirituality) OR "spiritual health" OR (spiritual* OR religion OR religiosity) OR (christian OR christianity OR islam OR muslim OR buddhism OR buddhist OR hindi OR hinduism OR jewish OR judaism OR sikh)) OR (transcend* OR "life purpose" OR "Existential needs" OR faith) MAINSUBJECT.EXACT("Faith")

AND (subject(mental health) OR tiab(mental wellness OR mental wellbeing OR depression OR anxiety OR stress)) AND ( "generation Z" OR "gen Z" OR youth OR "young adult" OR adolescent OR teenager OR "emerging adult" OR teen* OR "college students" OR "university students") AND (mhealth OR "digital health" OR "mobile app*" OR "mobile health" OR "digital intervention" OR ehealth OR telehealth OR "virtual medicine" OR telehealth OR "health app")

Filters - English only, 2003-2023

**Nov.7, 2021 = 23 results**

[mainsubject(spiritual well being) OR mainsubject(faith) OR mainsubject(spirituality) OR ("spiritual health" OR spiritual* OR religion OR religiosity OR Christian OR Christianity OR Islam OR Muslim OR Buddhism OR Buddhist OR Hindi OR Hinduism OR Jewish OR Judaism OR Sikh OR transcend* OR "life purpose" OR "Existential needs" OR faith OR spiritu* OR relig*)](https://www-proquest-com.ezproxy1.lib.asu.edu/recentsearches.recentsearchtabview.recentsearchesgridview.scrolledrecentsearchlist.checkdbssearchlink:rerunsearch/7F9649649F541ABPQ/None?site=psycinfo&t:ac=RecentSearches)

AND

[mhealth OR "digital health" OR "mobile app*" OR "mobile health" OR "digital intervention" OR ehealth OR telehealth OR "virtual medicine" OR telehealth OR "health app"](https://www-proquest-com.ezproxy1.lib.asu.edu/recentsearches.recentsearchtabview.recentsearchesgridview.scrolledrecentsearchlist.checkdbssearchlink:rerunsearch/2DE95FACE1847F4PQ/None?site=psycinfo&t:ac=RecentSearches)

AND

"generation z" OR "gen z" OR youth OR "Young adult" OR adolescent* OR teenager OR "emerging adult" OR teen* OR "college students" OR "University students"

AND

[subject(mental health) OR tiab(mental wellness OR mental wellbeing OR depression OR anxiety OR stress )](https://www-proquest-com.ezproxy1.lib.asu.edu/recentsearches.recentsearchtabview.recentsearchesgridview.scrolledrecentsearchlist.checkdbssearchlink:rerunsearch/AA10F2B6C7BF46B0PQ/None?site=psycinfo&t:ac=RecentSearches)

Filters - English only, 2003-2023

**CINAHL Plus with Full Text (EBSCO) = 6 results**

(MH "Mental Health") OR ( mental wellbeing or psychological wellbeing or emotional wellbeing or well being ) OR ( depression OR anxiety OR stress )

AND

( (MH "Religion and Religions") OR (MH "Judaism") OR (MH "Islam") OR (MH "Hinduism") ) OR ( Christianity OR Christian OR Sikh OR Buddhism OR Buddhist ) OR ( spirituality or religion )

AND

"gen z" OR "generation z" OR "post-millennials" OR teen* OR youth OR "emerging adult" OR "young adult" OR adolescence OR adolescent

AND

(MH "Telehealth") OR ( mhealth OR ehealth OR "mobile health" OR "health app" OR "digital health" OR "digital intervention" OR telehealth OR "virtual medicine" OR chatbot )

Updated search: September 26, 2023 = 11 results

(MH "Mental Health") OR ( mental wellbeing or psychological wellbeing or emotional wellbeing or well being ) OR ( depression OR anxiety OR stress )

AND

( (MH "Religion and Religions") OR (MH "Judaism") OR (MH "Islam") OR (MH "Hinduism") ) OR ( Christianity OR Christian OR Sikh OR Buddhism OR Buddhist ) OR ( spirituality or religion ) OR transcenden* OR "life purpose" OR "Existential needs" OR faith

AND

"gen z" OR "generation z" OR "post-millennials" OR teen* OR youth OR "emerging adult" OR "young adult" OR adolescence OR adolescent

AND

(MH "Telehealth") OR ( mhealth OR ehealth OR "mobile health" OR "health app" OR "digital health" OR "digital intervention" OR telehealth OR "virtual medicine" OR chatbot )

Filters: English only, results started in 2015

**Updated Search Nov.7, 2023 = 11 results**

MH mental health OR ( mental wellbeing OR mental well being OR psychological wellbeing OR emotional wellbeing OR depression OR anxiety OR stress )

AND

MH ( Religion and Religions ) OR MH Judaism OR MH islam OR MH Hinduism OR ( Christianity OR Christians OR Sikh OR Buddhism OR Buddhist OR Jewish OR Muslim OR Hindi ) OR ( spirituality or religion or faith or belief system OR transcenden* OR "life purpose" OR "existential needs" OR faith OR Spiritu* OR Relig* )

AND

"gen z" OR "generation z" OR "post-millennials" OR teen* OR youth OR "emerging adult" OR "young adult" OR adolescence OR adolescent

AND

MH telehealth OR ( telemedicine OR mhealth OR ehealth OR "mobile health" OR "health app" OR "digital health" OR "digital intervention" OR telehealth OR "virtual medicine" OR chatbot )

Filter: English only, self limited to 2015 - did not download results as they were the same as Sept search

**Google Scholar - download first 50 results**

mhealth OR "digital health" OR "mobile app" OR "mobile health" OR "digital intervention" OR ehealth OR telehealth OR "virtual medicine" OR telehealth OR "health app" AND "gen z" OR "generation Z" OR youth OR teen* OR "young adult" OR "emerging adult" OR "college students" OR "university students" OR adolescent* OR adolescence AND "mental health" OR wellness OR wellbeing OR depression OR anxiety OR stress AND spirituality OR spiritual OR religiosity OR religion OR christian OR christianity OR islam OR muslim OR buddhism OR buddhist OR hindi OR hinduism OR jewish OR judaism OR sikh OR faith

Updated search Sept. 26, 20223 = downloaded first 40 results by relevance

mhealth OR "digital health" OR "mobile app" OR "mobile health" OR "digital intervention" OR ehealth OR telehealth OR "virtual medicine" OR telehealth OR "health app" AND "gen z" OR "generation Z" OR youth OR teen* OR "young adult" OR "emerging adult" OR "college students" OR "university students" OR adolescent* OR adolescence AND "mental health" OR wellness OR wellbeing OR depression OR anxiety OR stress AND spirituality OR spiritual OR religiosity OR religion OR christian OR christianity OR islam OR muslim OR buddhism OR buddhist OR hindi OR hinduism OR jewish OR judaism OR sikh OR transcenden* OR "life purpose" OR "Existential needs" OR faith

**Updated search Nov.8, 2023** = first 100 citations downloaded - 61 were new

(mhealth OR "digital health" OR "mobile app" OR "mobile health" OR "digital intervention" OR ehealth OR telehealth OR "virtual medicine" OR telehealth OR "health app") AND ("gen z" OR "generation Z" OR youth OR teen* OR "young adult" OR "emerging adult" OR "college students" OR "university students" OR adolescent* OR adolescence) AND ("mental health" OR wellness OR wellbeing OR depression OR anxiety OR stress) AND (spiritu* OR relig* OR christian OR christianity OR islam OR muslim OR buddhism OR buddhist OR hindi OR hinduism OR jewish OR judaism OR sikh OR transcenden* OR "life purpose" OR "Existential needs" OR faith)

**ERIC - 0 results**

mhealth OR "digital health" OR "mobile app" OR "mobile health" OR "digital intervention" OR ehealth OR telehealth OR "virtual medicine" OR telehealth OR "health app"

AND

"gen z" OR "generation Z" OR youth OR teen* OR "young adult" OR "emerging adult" OR "college students" OR "university students" OR adolescent* OR adolescence

AND

"mental health" OR wellness OR wellbeing OR depression OR anxiety OR stress

AND

spirituality OR spiritual OR religiosity OR religion OR christian OR christianity OR islam OR muslim OR buddhism OR buddhist OR hindi OR hinduism OR jewish OR judaism OR sikh OR transcenden* OR "life purpose" OR "Existential needs" OR faith

Updated search Sept.26, 2023 still 0 results

**Updated search Nov.7, 20203 = still 0 results**

mhealth OR "digital health" OR "mobile app" OR "mobile health" OR "digital intervention" OR ehealth OR telehealth OR "virtual medicine" OR telehealth OR "health app"

AND

"gen z" OR "generation Z" OR youth OR teen* OR "young adult" OR "emerging adult" OR "college students" OR "university students" OR adolescent* OR adolescence

AND

"mental health" OR wellness OR wellbeing OR depression OR anxiety OR stress

AND

spirituality OR spiritual OR religiosity OR religion OR Christian OR Christianity OR Islam OR Muslim OR Buddhism OR Buddhist OR Hindi OR Hinduism OR Jewish OR Judaism OR Sikh OR transcenden* OR "life purpose" OR "Existential needs" OR faith OR spiritu* OR relig*

**Education Full Text (H.W. Wilson) (EBSCO) = 2 results**

mhealth OR "digital health" OR "mobile app" OR "mobile health" OR "digital intervention" OR ehealth OR telehealth OR "virtual medicine" OR telehealth OR "health app"

AND

"gen z" OR "generation Z" OR youth OR teen* OR "young adult" OR "emerging adult" OR "college students" OR "university students" OR adolescent* OR adolescence

AND

"mental health" OR wellness OR wellbeing OR depression OR anxiety OR stress

AND

spirituality OR spiritual OR religiosity OR religion OR christian OR christianity OR islam OR muslim OR buddhism OR buddhist OR hindi OR hinduism OR jewish OR judaism OR sikh

Updated search September 26, 2023 = 7 results

mhealth OR "digital health" OR "mobile app" OR "mobile health" OR "digital intervention" OR ehealth OR telehealth OR "virtual medicine" OR telehealth OR "health app"

AND

"gen z" OR "generation Z" OR youth OR teen* OR "young adult" OR "emerging adult" OR "college students" OR "university students" OR adolescent* OR adolescence

AND

"mental health" OR wellness OR wellbeing OR depression OR anxiety OR stress

AND

spirituality OR spiritual OR religiosity OR religion OR christian OR christianity OR islam OR muslim OR buddhism OR buddhist OR hindi OR hinduism OR jewish OR judaism OR sikh OR transcenden* OR "life purpose" OR "Existential needs" OR faith

Filters: English only, self selected back to 2017

**Updated Search Nov.7, 2023 = 7 results (same number as Sept, did not download results)**

mhealth OR "digital health" OR "mobile app" OR "mobile health" OR "digital intervention" OR ehealth OR telehealth OR "virtual medicine" OR telehealth OR "health app"

AND

"gen z" OR "generation Z" OR youth OR teen* OR "young adult" OR "emerging adult" OR "college students" OR "university students" OR adolescent* OR adolescence

AND

"mental health" OR wellness OR wellbeing OR depression OR anxiety OR stress

AND

spirituality OR spiritual OR religiosity OR religion OR Christian OR Christianity OR Islam OR Muslim OR Buddhism OR Buddhist OR Hindi OR Hinduism OR Jewish OR Judaism OR Sikh OR transcenden* OR "life purpose" OR "Existential needs" OR faith OR spiritu* OR relig*

**SocINDEX with Full Text (EBSCO) = 3 results**

mhealth OR "digital health" OR "mobile app" OR "mobile health" OR "digital intervention" OR ehealth OR telehealth OR "virtual medicine" OR telehealth OR "health app"

AND

"gen z" OR "generation Z" OR youth OR teen* OR "young adult" OR "emerging adult" OR "college students" OR "university students" OR adolescent* OR adolescence

AND

"mental health" OR wellness OR wellbeing OR depression OR anxiety OR stress

AND

spirituality OR spiritual OR religiosity OR religion OR christian OR christianity OR islam OR muslim OR buddhism OR buddhist OR hindi OR hinduism OR jewish OR judaism OR sikh OR faith

Updated search Sept.26, 2023 = 4 results

mhealth OR "digital health" OR "mobile app" OR "mobile health" OR "digital intervention" OR ehealth OR telehealth OR "virtual medicine" OR telehealth OR "health app"

AND

"gen z" OR "generation Z" OR youth OR teen* OR "young adult" OR "emerging adult" OR "college students" OR "university students" OR adolescent* OR adolescence

AND

"mental health" OR wellness OR wellbeing OR depression OR anxiety OR stress

AND

spirituality OR spiritual OR religiosity OR religion OR christian OR christianity OR islam OR muslim OR buddhism OR buddhist OR hindi OR hinduism OR jewish OR judaism OR sikh OR transcenden* OR "life purpose" OR "Existential needs" OR faith

Filters: English only, self selected to 2018

**Updated Search Nov.7, 2023 = 4 results** (did not download as numbers did not change)

mhealth OR "digital health" OR "mobile app" OR "mobile health" OR "digital intervention" OR ehealth OR telehealth OR "virtual medicine" OR telehealth OR "health app"

AND

"gen z" OR "generation Z" OR youth OR teen* OR "young adult" OR "emerging adult" OR "college students" OR "university students" OR adolescent* OR adolescence

AND

"mental health" OR wellness OR wellbeing OR depression OR anxiety OR stress

AND

spirituality OR spiritual OR religiosity OR religion OR Christian OR Christianity OR Islam OR Muslim OR Buddhism OR Buddhist OR Hindi OR Hinduism OR Jewish OR Judaism OR Sikh OR transcenden* OR "life purpose" OR "Existential needs" OR faith OR spiritu* OR relig*

Filters: English only, self selected to 2018

**Sociological Abstracts (ProQuest) = 155, imported first 100 into Covidence by relevance**

mhealth OR "digital health" OR "mobile app" OR "mobile health" OR "digital intervention" OR ehealth OR telehealth OR "virtual medicine" OR telehealth OR "health app"

AND

"gen z" OR "generation Z" OR youth OR teen* OR "young adult" OR "emerging adult" OR "college students" OR "university students" OR adolescent* OR adolescence

AND

"mental health" OR wellness OR wellbeing OR depression OR anxiety OR stress

AND

spirituality OR spiritual OR religiosity OR religion OR christian OR christianity OR islam OR muslim OR buddhism OR buddhist OR hindi OR hinduism OR jewish OR judaism OR sikh OR faith

- Added filter for “Scholarly articles”

Updated Search September 27, 2023 = 279 results. Imported first 100 by relevance

mhealth OR "digital health" OR "mobile app" OR "mobile health" OR "digital intervention" OR ehealth OR telehealth OR "virtual medicine" OR telehealth OR "health app"

AND

"gen z" OR "generation Z" OR youth OR teen* OR "young adult" OR "emerging adult" OR "college students" OR "university students" OR adolescent* OR adolescence

AND

"mental health" OR wellness OR wellbeing OR depression OR anxiety OR stress

AND

spirituality OR spiritual OR religiosity OR religion OR christian OR christianity OR islam OR muslim OR buddhism OR buddhist OR hindi OR hinduism OR jewish OR judaism OR sikh OR faith OR transcenden* OR "life purpose" OR "Existential needs"

Filters used: English only, Scholarly articles, self selected back to 1995

**Updated Search Nov.7, 2023 = 315 results (first 100 by relevance downloaded)**

mhealth OR "digital health" OR "mobile app" OR "mobile health" OR "digital intervention" OR ehealth OR telehealth OR "virtual medicine" OR telehealth OR "health app"

AND

"gen z" OR "generation Z" OR youth OR teen* OR "young adult" OR "emerging adult" OR "college students" OR "university students" OR adolescent* OR adolescence

AND

"mental health" OR wellness OR wellbeing OR depression OR anxiety OR stress

AND

spirituality OR spiritual OR religiosity OR religion OR Christian OR Christianity OR Islam OR Muslim OR Buddhism OR Buddhist OR Hindi OR Hinduism OR Jewish OR Judaism OR Sikh OR transcenden* OR "life purpose" OR "Existential needs" OR faith OR spiritu* OR relig*

Filter: English only. Self selected to 1995

**ATLA Religion Database with Atla Serials (EBSCO) = 0 results**

mhealth OR "digital health" OR "mobile app" OR "mobile health" OR "digital intervention" OR ehealth OR telehealth OR "virtual medicine" OR telehealth OR "health app"

AND

"gen z" OR "generation Z" OR youth OR teen* OR "young adult" OR "emerging adult" OR "college students" OR "university students" OR adolescent* OR adolescence

AND

"mental health" OR wellness OR wellbeing OR depression OR anxiety OR stress

AND

spirituality OR spiritual OR religiosity OR religion OR christian OR christianity OR islam OR muslim OR buddhism OR buddhist OR hindi OR hinduism OR jewish OR judaism OR sikh OR faith OR transcenden* OR "life purpose" OR "Existential needs"

Updated search Sept.26, 2023 = 0 results

**Updated search Nov.7, 2023** = 0 results

mhealth OR "digital health" OR "mobile app" OR "mobile health" OR "digital intervention" OR ehealth OR telehealth OR "virtual medicine" OR telehealth OR "health app"

AND

"gen z" OR "generation Z" OR youth OR teen* OR "young adult" OR "emerging adult" OR "college students" OR "university students" OR adolescent* OR adolescence

AND

"mental health" OR wellness OR wellbeing OR depression OR anxiety OR stress

AND

spirituality OR spiritual OR religiosity OR religion OR Christian OR Christianity OR Islam OR Muslim OR Buddhism OR Buddhist OR Hindi OR Hinduism OR Jewish OR Judaism OR Sikh OR transcenden* OR "life purpose" OR "Existential needs" OR faith OR spiritu* OR relig*
